# Economic burden of multidrug-resistant tuberculosis on patients and households: a global systematic review and meta-analysis

**DOI:** 10.1038/s41598-023-47094-9

**Published:** 2023-12-15

**Authors:** Temesgen Yihunie Akalu, Archie C. A. Clements, Haileab Fekadu Wolde, Kefyalew Addis Alene

**Affiliations:** 1 Faculty of Health Sciences, Curtin University, Perth, WA Australia; 2 Geospital and Tuberculosis Research Team, Telethon Kids Institute, Perth, WA Australia; 3 Institute of Public Health, College of Medicine and Health Sciences, University of Gondar, Gondar, Ethiopia; 4 Peninsula Medical School, University of Plymouth, Plymouth, UK

**Keywords:** Medical research, Epidemiology, Tuberculosis

## Abstract

Multidrug-resistant tuberculosis (MDR-TB) is a major health threat worldwide, causing a significant economic burden to patients and their families. Due to the longer duration of treatment and expensive second-line medicine, the economic burden of MDR-TB is assumed to be higher than drug-susceptible TB. However, the costs associated with MDR-TB are yet to be comprehensively quantified. We conducted this systematic review and meta-analysis to determine the global burden of catastrophic costs associated with MDR-TB on patients and their households. We systematically searched five databases (CINHAL, MEDLINE, Embase, Scopus, and Web of Science) from inception to 2 September 2022 for studies reporting catastrophic costs on patients and affected families of MDR-TB. The primary outcome of our study was the proportion of patients and households with catastrophic costs. Costs were considered catastrophic when a patient spends 20% or more of their annual household income on their MDR-TB diagnosis and care. The pooled proportion of catastrophic cost was determined using a random-effects meta-analysis. Publication bias was assessed using visualization of the funnel plots and the Egger regression test. Heterogeneity was assessed using I^2^, and sub-group analysis was conducted using study covariates as stratification variables. Finally, we used the Preferred Reporting Items for Reporting Systematic Review and Meta-Analysis-20 (PRISMA-20). The research protocol was registered in PROSPERO (CRD42021250909). Our search identified 6635 studies, of which 11 were included after the screening. MDR-TB patients incurred total costs ranging from $USD 650 to $USD 8266 during treatment. The mean direct cost and indirect cost incurred by MDR-TB patients were $USD 1936.25 (SD ± $USD 1897.03) and $USD 1200.35 (SD ± $USD 489.76), respectively. The overall burden of catastrophic cost among MDR-TB patients and households was 81.58% (95% Confidence Interval (CI) 74.13–89.04%). The catastrophic costs incurred by MDR-TB patients were significantly higher than previously reported for DS-TB patients. MDR-TB patients incurred more expenditure for direct costs than indirect costs. Social protection and financial support for patients and affected families are needed to mitigate the catastrophic economic consequences of MDR-TB.

## Introduction

Multidrug-resistant tuberculosis (MDR-TB) is a major health threat globally, affecting nearly half a million people in 2020^1^. MDR-TB disproportionately affects the most vulnerable populations in low- and middle-income countries^2,3^. Ending catastrophic costs in TB-affected households is one of the targets of the World Health Organization (WHO) end-TB strategy^4^. However, the widespread dissemination of MDR- and Extensively drug-resistant (XDR)-TB continues to be a significant obstacle to achieving these ambitious targets^5^.

The WHO defines catastrophic TB costs as total (direct and indirect) costs of TB diagnosis and care above 20% of the household’s annual income^6^. Direct cost includes medical (registration, consultation, hospitalization, investigation, or medication) and non-medical (food, travel, and nutritional supplements). Indirect costs include overall loss of wages due to productivity loss, missed work, loss of time, loss of income, and caregiving work^7^. In times of financial hardship, TB patients or TB-affected households implement different coping strategies, such as selling household assets, borrowing money, utilizing savings, and taking children out of school^8^.

Due to the longer duration of treatment and the need for expensive second-line medications, the catastrophic costs associated with MDR-TB are thought to be higher than the catastrophic costs associated with drug-susceptible TB (DS-TB)^9^. The economic consequences of MDR-TB are often so severe that patients and affected families can fall into extreme poverty because of high out-of-pocket expenses and income lost during MDR-TB diagnosis and treatment^10,11^. Studies have shown that MDR-TB patients face considerable financial losses before, during, and after treatment^12,13^. For instance, a systematic review conducted in several countries showed that the cost of treatment among MDR-TB patients ranges from 2423 United States Dollars (USD) in Peru to USD 14,657 in Tomsk, Russia^14^.

The burden of catastrophic cost among MDR-TB patients is affected by different factors, including sociodemographic factors, diagnosis delay, length of hospital stay, household wealth status, distance from the health facilities, number of household members, health care setting (private vs public), pre-TB expenditures, and hospitalizations^15,16^. A systematic review conducted among TB patients (DS- and DR-TB combined) showed that the proportion of catastrophic costs was 43%^17^. However, the burden of catastrophic costs among MDR-TB patients is expected to be higher because of the longer duration of treatment needed and the high cost of MDR-TB medicines compared with DS-TB patients^18^. However, the costs associated with MDR-TB are yet to be comprehensively quantified. We conducted this systematic review and meta-analysis to determine the global burden of catastrophic costs associated with MDR-TB on patients and their households.

## Methods

This protocol is reported following the Preferred Reporting Items for Systematic Review and Meta-Analysis (PRISMA) guidelines^19^, which are detailed in supplementary file S1. The protocol is published in the PROSPERO, with a registration number CRD42021250909.

### Searching strategy and eligibility criteria

Systematic searches from five databases (CINAHL (EBSCO), MEDLINE (via Ovid), Embase, Scopus, and Web of Science) were conducted to retrieve relevant studies. We also searched the grey literature using Google and Google Scholar. Reference lists of included studies were searched for additional relevant articles. The search terms included “multidrug-resistant tuberculosis” (multidrug-resistant tuberculosis, MDR-TB), “extensively drug-resistant tuberculosis” (extensively drug-resistant tuberculosis, XDR-TB), and “cost” (cost(s), loss, sales, loan, economics, finance, expense, expenditure, payment, and impoverishment). The search strategy for all databases is provided in supplementary table 2. The search was performed from the inception of each database to 2 September 2022.

### Study selection criteria

All potential publications reporting direct or indirect costs incurred by MDR-TB patients and their families and reporting percentages of catastrophic costs were included. Direct costs include direct medical costs (hospitalization, investigation, medication, and registration/consultation) and direct nonmedical costs (food, travel, and special diet). Indirect costs include income loss due to MDR-TB patients and caregivers absent from their jobs^20^. We excluded conference abstracts, animal studies, case studies, reviews, studies that report only on DS-TB, and studies that did not provide data on catastrophic costs. We also excluded studies that reported only medication cost, hospitalization cost, investigation cost, and cost data from facilities, program, and health system perspectives. However, there were no restrictions to non-English language articles. Google Translate was used to translate non-English language articles.

### Outcomes of the study

The primary outcome was the proportion of patients with MDR-TB or their households who incurred catastrophic costs. The secondary outcomes of the review were the proportion of the total costs that were direct costs of MDR-TB treatment among MDR-TB patients and the proportion of individuals resorting to different coping mechanisms. Coping mechanisms include borrowing money, selling assets, and utilization of savings. All costs were reported in USD.

### Study selection, data extraction, and quality assessment

After the search, all identified articles were imported into an EndNote version 7 (Thomson Reuters, London) library. Then, duplicated citations were identified and removed. After removing duplication, citations were exported to Rayyan software (Rayyan, Cambridge) for further screening by title and abstract. Two independent reviewers (TYA and HFW) screened the titles and abstracts and reviewed the full text to identify relevant studies. When there were differences between the two authors, a decision was made by consensus. Data were extracted from included studies using a Microsoft Excel (version 2014) spreadsheet on the following information: (1) study characteristics including the name of the first author, year of publication, country of the study, study setting (urban vs rural), and study design, (2) participant's characteristics such as study population (i.e. MDR-TB, XDR-TB, or both), mean or median age, the proportion of male, and sample size; (3) clinical characteristics such as type of MDR-TB medications, duration of treatments, and comorbidity (HIV, diabetes Mellitus); and (4) outcomes of the study, including catastrophic cost and disaggregation of costs. The Newcastle Ottawa Scale (NOS) was used to assess the quality of included studies. The NOS judges the quality of observational studies with three basic perspectives: selection of study groups (4 points), comparability of groups (2 points), and ascertainment of the outcome of interest (3 points). The overall score ranges from zero to nine, with low (0–4), medium (5–7), and high (8–9) quality groupings^21,22^.

### Data synthesis

The choice of a fixed-effect or random-effect meta-analysis model is determined by the presence of heterogeneity. In our case, we used a random-effects meta-analysis to determine the proportions of catastrophic costs among MDR-TB patients due to a significant level of heterogeneity among the included studies with I^2^ value > 90%. The random-effects meta-analysis assumes the effect size may vary among studies, and it also addresses both the intra-study variation (sampling error) and inter-study variation.

The proportion of direct and indirect costs was reported. The pooled proportion of catastrophic costs was determined using a forest plot. The presence of heterogeneity between included studies was assessed using Cochran’s Q test and measured quantitatively by the index of heterogeneity squared (I^2^), with 95% Confidence Interval (CI)^23^. The level of heterogeneity was classified as low (I^2^ =  < 25%), moderate (I^2^ = between 25 and 75%) or high (I^2^ =  > 75%). In the presence of heterogeneity, sub-group analysis was conducted to assess the risk of catastrophic costs among groups having adequate and comparable data. The country’s income, study design, year of data collection, type of study population, HIV status, and data collection tool were used for sub-group analysis. Publication bias was reported using visualization of the funnel plot and the Egger regression test^24^. STATA version 17 software was used to conduct the analysis. All costs were initially presented in US dollars. Sensitivity analysis was conducted by removing low-quality studies.

### Role of funder

The study was funded by the Australian National Health and Medical Research Council through an Emerging Leadership Investigator grant and a Curtin University Higher Degree Research scholarship. The funders had no role in study design, data collection, analysis, interpretation, or report writing. The corresponding author had full access to all data in the study. ACAC and KAA were ultimately responsible for the decision to submit for publication.

### Ethics approval

Since we used publicly available published data, ethical approval was not required.

## Results

### Characteristics of included studies

**Figure 1:**
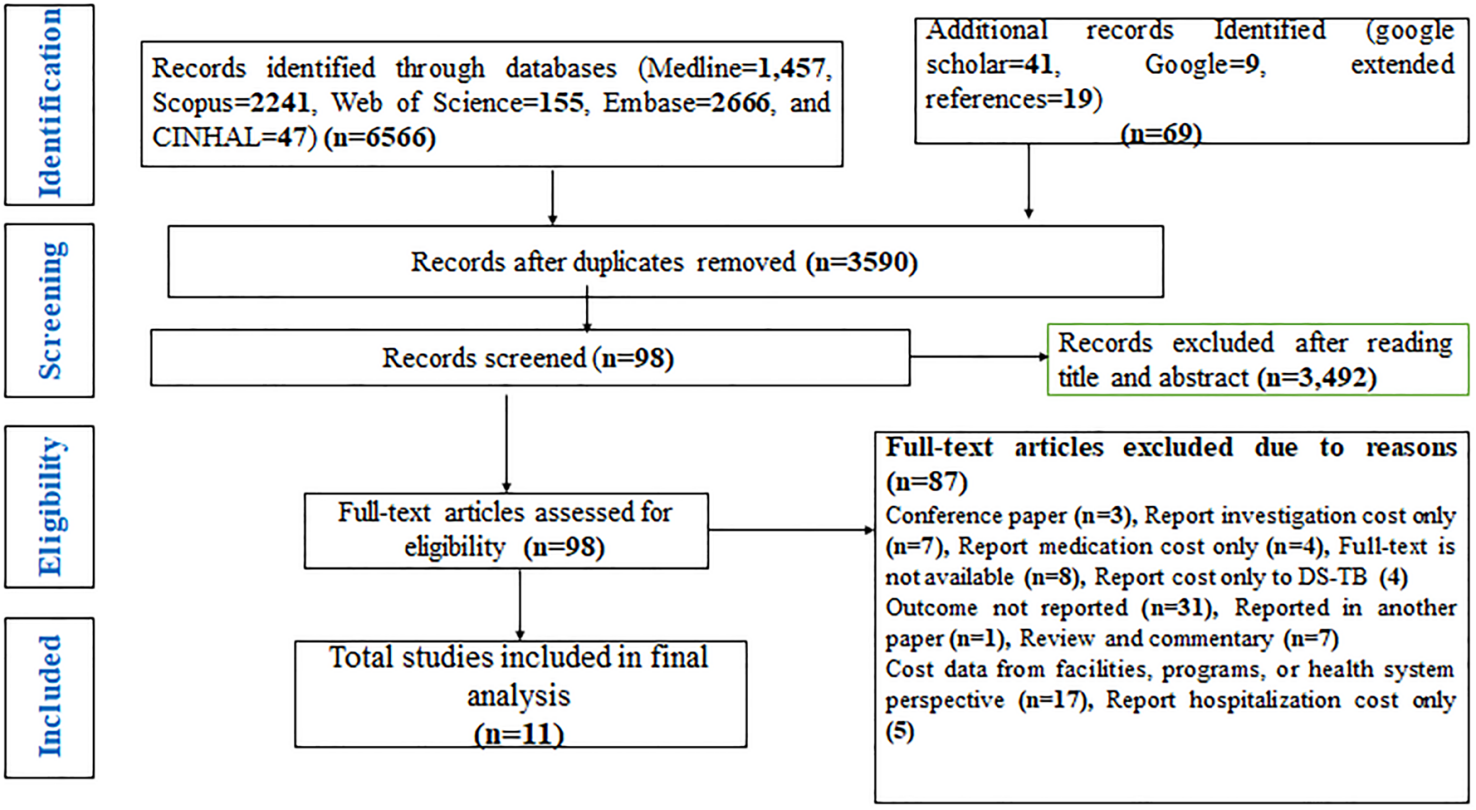
Study selection process of the meta-analysis.

The search strategy identified 6635 potential studies (6566 from electronic databases and 69 using additional records) for evaluation. After duplicates were removed, 3590 citations were screened by title and abstract, and 98 eligible articles had a full-text review. In total, 11 articles^16,25–34^ that provided data on 817 MDR-TB patients were included in the meta-analysis. The overall study selection process and reasons for exclusion are presented in Fig. 1.

The included studies were conducted in 11 different countries (seven studies from Low-Middle Income Countries (LMICs), three studies were from Low-Income Countries (LICs) countries, and one study was from an Upper Middle-Income Countries (UMICs)), and the data were collected from 2002 to 2019 (Table 1). The monthly income of households was reported in three studies, and the value ranged from $USD 154.47 in Ghana to $USD 418 in Peru. Self-prepared tools were used to measure catastrophic costs in two included studies, and the other nine used the WHO TB cost survey tool (Table 1).

**Table 1:** Characteristics of included studies.

First author	Publication year	Country	Country income	Year of data collection	Study design	Male proportion	Mean age	Sample size	HIV prevalence (%)	Tool used	Treatment regimen
Nhung	2018	Viet Nam	LMI	2016	Cross-sectional	81	41	58	5.1	WHO adapted tool	Long-term
Collins	2021	Zimbabwe	LMI	2018	Cross-sectional	56	36.4	49	72	WHO adapted tool	NR
Phonenaly		Laos	LMI	2018	Cross-sectional	87.5	41.4	8	0	WHO adapted tool	Short-term
Yun	2020	China	UMI	2018	Cross-sectional	68.9	36	161	NR	Prepared locally	NR
Ahmad	2018	Indonesia	LMI	2016	Cross-sectional	48	NR	64	0	Bahasa Indonesia version	NR
Winters	2020	Uganda	LI	2017	Cross-sectional	67.9	NR	44	57.3	WHO TB cost survey	Long-term
Kaswa	2021	DR. Congo	LI	2019	Cross-sectional	61.9	NR	202	16.8	WHO adapted tool	Long-term
Tomeny	2020	Philippines	LMI	2016	Cross-sectional		NR	25	NR	WHO TB cost survey	NR
Andrew	2022	Tanzania	LMI	2019	Cross-sectional	84	42.5	25	NR	WHO TB cost survey	NR
Debora	2018	Ghana	LMI	2016	Cross-sectional	63.6	43	66	12.1	WHO adapted tool	NR
Tom	2014	Peru	UMI	2002–2009	Prospective cohort	59	31	93		Prepared locally	NR

### Cost of MDR-TB treatment

**Figure 2:**
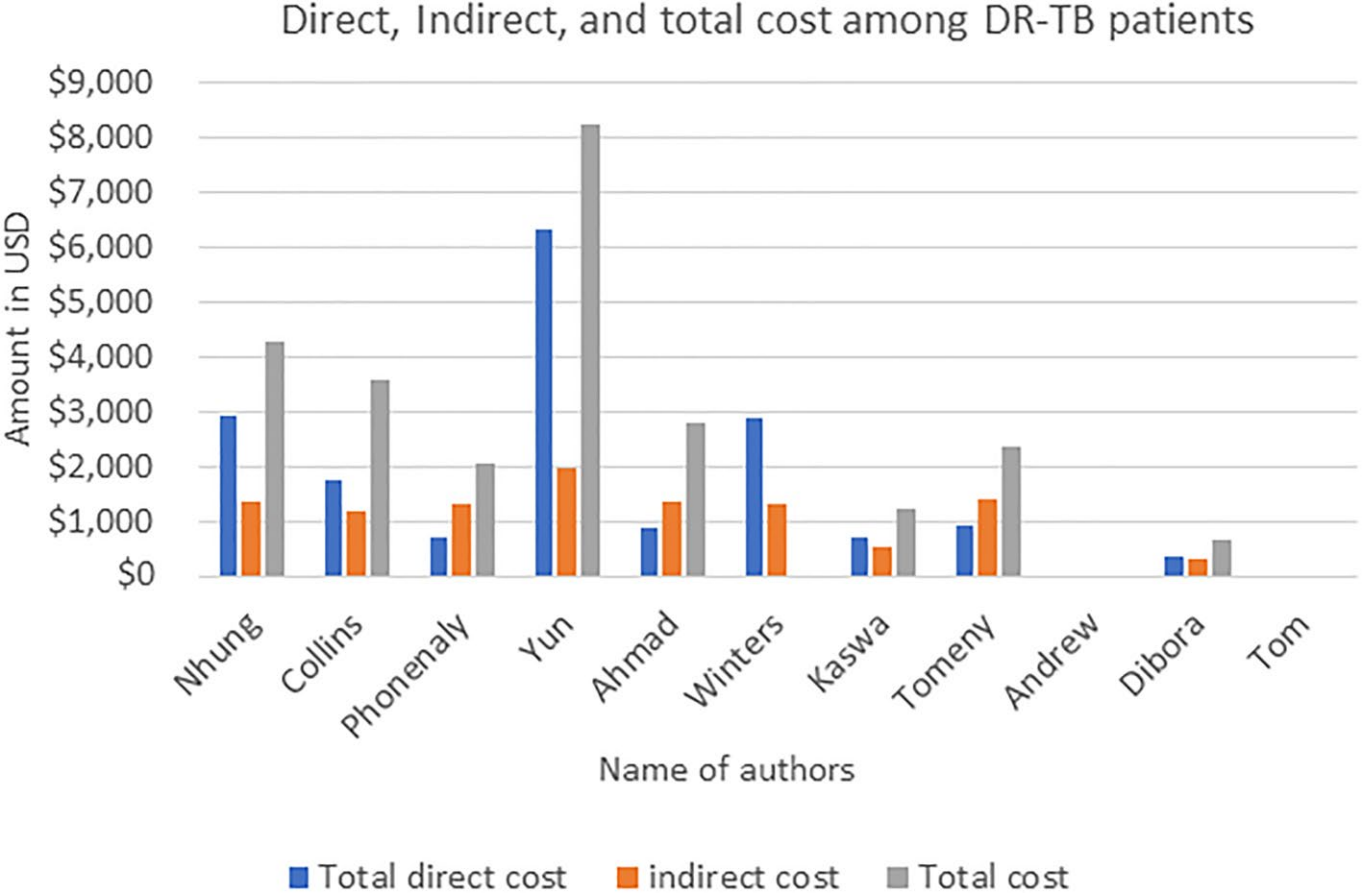
Direct, indirect, and total costs incurred by MDR-TB patients for their treatment.

Nine studies^16,25–30,32,33^ reported a detailed cost breakdown for treating MDR-TB. From the nine studies, five studies^25–27,29,33^ reported cost breakdowns for direct and indirect medical costs. The mean direct cost incurred by MDR-TB patients was $USD 1,936.25 (SD ± $USD 1897.03). The mean indirect cost incurred for the treatment of MDR-TB was $USD 1,200.35 (SD ± $USD 489.76). The mean total payment incurred by MDR-TB patients was $USD 3,151.19 (SD ± $USD 2379.15). The direct (medical and non-medical) and indirect costs are summarized in Table 2 and Fig. 2.

**Table 2:** Direct cost and indirect cost incurred for the treatment of MDR-TB patients and its coping mechanisms.

Authors (publication year)	Direct medical cost				Direct non-medical cost					Total direct cost	Indirect cost			Total cost
	Hospitalization	Medication	Others**	Total cost	Food	travel	Special diet	Others***	Total cost		Productivity loss	Loss income	Caregiver cost	
Nhung (2018)	**–**	**–**	**–**	791*	**–**	**–**	**–**		2134*	2,925	1,376	**–**	**–**	4,302
Collins (2021)	**–**	**–**	**–**	220	109	157	962	317	1545	1,765	1,200	**–**	**–**	3,569
Phonenaly 2020	214	203		417	33	92	161	10	296	713	1,324	**–**	**–**	2,037
Yun 2020				5934*	**–**	**–**	**–**		382*	6,316	1,950			8,266
Ahmad 2018	29			29	269	403	179		851	880		1,344		2,804
Winters 2020			56	79.3	498.7	1,025.5	1,263.4		2787.64	2,866.94	1,219		115	4,200.94
Kaswa 2021	15.1		119.2	134	184.1	296.5	76.1		556.7	691	533			1,224
Tomeny 2020		59.2		59.2	70.3	464.2	344.15		878.65	937.85	1384.6			2,357
Andrew (2022)	**–**	**–**	**–**	**–**	**–**	**–**	**–**	**–**	**–**	**–**	**–**	**–**	**–**	**–**
Debora (2018)				65	234	32.5			266	331.5	318.5			650
Tom (2014)	**–**	**–**	**–**	**–**	**–**	**–**	**–**	**–**	**–**	**–**	**–**	**–**	**–**	**–**
Mean with SD										**1936.25 ± 1897.03**	**1200.35 ± 489.76**			**3151.19 ± 2379.15**

### Catastrophic costs among MDR-TB patients

**Figure 3:**
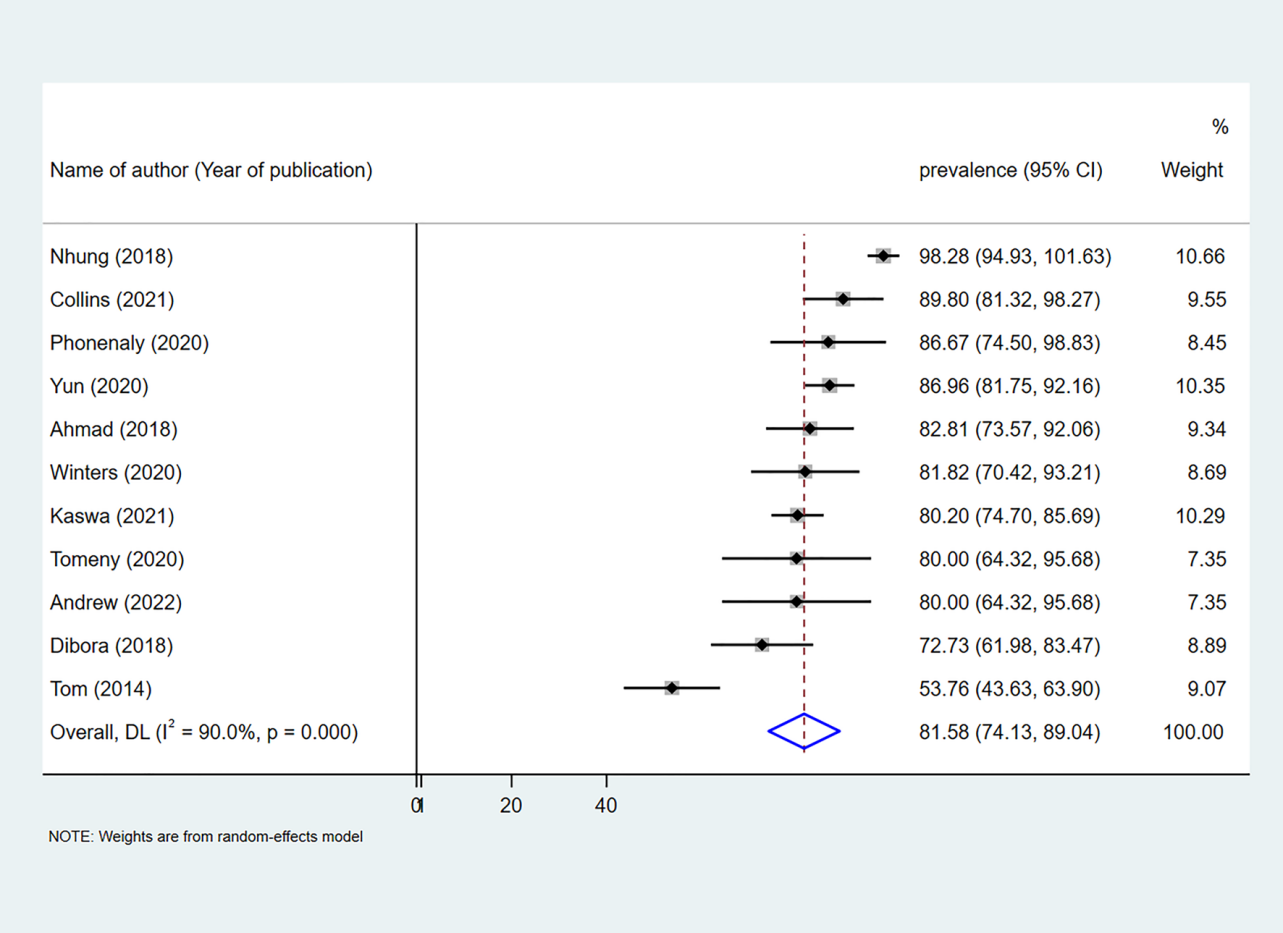
Pooled proportion of catastrophic cost among both patients and households.

All the studies define catastrophic cost at a 20% cut-off point. Based on this definition, the overall pooled percentages of catastrophic costs among MDR-TB patients and households were 81.58% (95% CI 74.13–89.04%) with I^2^ of 90.0%, indicating considerable heterogeneity of effect estimates between studies (Fig. 3). The proportion of catastrophic costs among MDR-TB patients ranged from 53.76% in Peru to 98.28% in Viet Nam.

### Sub-group analysis

The burden of catastrophic costs among MDR-TB patients varied by country income level, with the highest burden of catastrophic costs reported in LMICs (85.18%; 95% CI 76.81–93.55) and the lowest burden reported in UMICs (70.66%; 95% CI 38.13–103.18) with I^2^ = 96.9%, p = 0.000. The burden of catastrophic cost also varied by HIV status, in which catastrophic cost was significantly higher in studies with a higher prevalence (i.e., above the median value of 50%) of HIV (86.75%; 95% CI 79.16–94.35%) than in studies with a lower HIV prevalence (84.52%; 95% CI; 73.81–95.23) with I^2^ = 82%, p = 0.000 (Table 3).

**Table 3:** Burden of catastrophic cost stratified by country income, HIV status, time of data collection, a tool used for data collection, and study design.

Stratification by	Number of studies	Proportion (95% CI)	DL (I^2^) with a p-value
Country income level LICs LMICs UMICs	2 7 2	**80.50 (75.55, 85.45)** 85.18 (76.81, 93.55) 70.66 (38.13, 103.18)	90%, p = 0.000 82.7%, p = 0.000 96.9%, p = 0.000
HIV status < 50% ≥ 50% Not reported	5 2 4	84.52 (73.81, 95.23) **86.75 (79.16, 94.35)** 75.12 (57.64, 92.60)	91.6%, p = 0.000 17.5%, p = 0.271 90.8%, p = 0.000
Time of data collection 2002–2009 2016 2017 2018 2019	1 4 1 1 4	53.76 (43.63, 63.90) 84.15 (70.41, 97.88) 81.82 (70.42, 93.21) 87.61 (83.44, 91.77) 80.18 (74.99, 85.36)	0%, p = 90%, p = 0.000 0%, p = 0%, p = 0.844 0%, p = 0.981
A tool used for data collection Adapted from the WHO tool Prepared locally Adapted Bahsha Indonesia version WHO TB cost survey	5 2 1 3	85.94 (75.74, 96.15) 70.66 (38.13, 103.18) 82.81 (73.57, 92.06) 80.88 (72.94, 88.83)	91.0%, p = 0.000 96.9%, p = 0.000 0%, p = 0%, p = 0.975
Study design Cross-sectional Cohort	10 1	84.64 (78.56, 90.73) 53.76 (43.63, 63.90)	83.5, p = 0.000 0, p =

### Coping mechanisms

Three studies^25,29,31^ reported different coping mechanisms to alleviate out-of-pocket expenses during MDR-TB treatment. The most used coping mechanisms were borrowing, selling assets, and utilizing savings. The remaining eight studies did not report any forms of coping mechanisms.

### Quality and publication bias assessment

**Figure 4:**
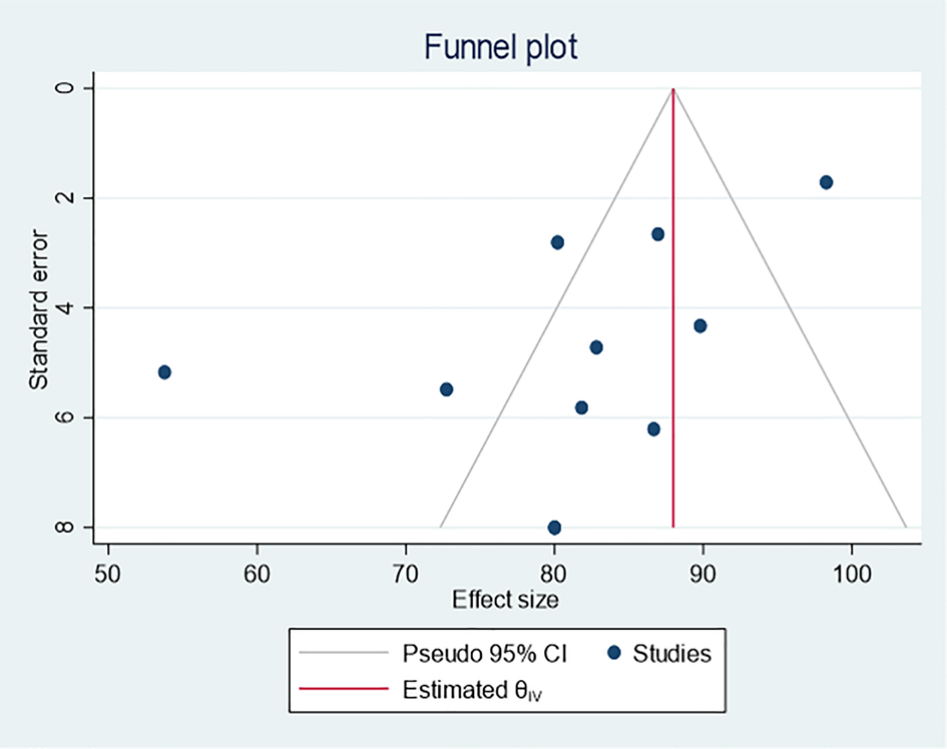
Funnel plot indicating the presence of publication bias.

The quality of included studies ranged from low to high, with a median score of 4 points and a maximum score of 8 points. Of the included 11 studies, there is no study, regarded as a good-quality study. Five studies had a score of five to seven points, regarded as moderate-quality studies, and the remaining six studies had a score of less than five points, regarded as low-quality studies (Supplementary file 3). There was evidence of publication bias in the statistical and visual appraisal of funnel plots (Fig. 4).

### Sensitivity analysis

**Figure 5:**
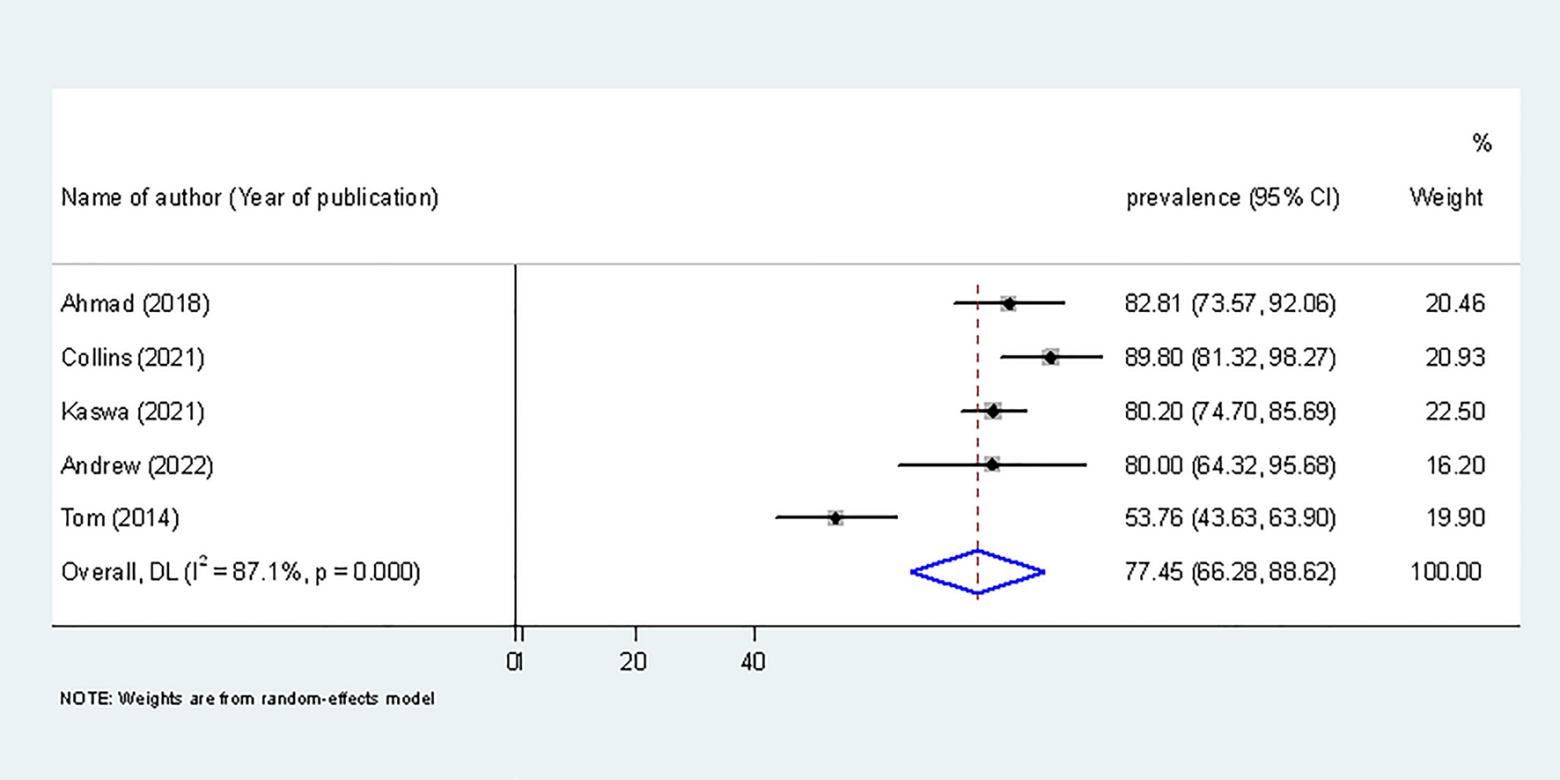
Sensitivity analysis to show the burden of catastrophic cost by removing low-quality studies.

The burden of catastrophic cost after removing low-quality studies was 77.45% (95% CI 66.28–88.62) with I^2^ = 87.1%, p = 0.000 (Fig. 5). However, the finding showed no difference from the original that includes all studies (81.58%; 95% CI 74.13–89.05).

## Discussion

To our knowledge, this systematic review and meta-analysis is the most comprehensive data synthesis reported to date, presenting the economic burden of MDR-TB faced by patients and affected households at a global level. Our systematic review identified only 11 eligible studies reporting the economic consequences of MDR-TB, including 817 MDR-TB patients with available data. This finding indicates that studies quantifying the economic impacts of MDR-TB remain inadequate despite the growing burden of MDR-TB, and more data are urgently needed to inform options for mitigating the economic consequences of MDR-TB on patients and their families. The proportion of catastrophic cost, as measured by the proportion of patients with total costs exceeding 20% of their annual household income, was almost double in MDR-TB (81.58%) than in DS-TB (43%) patients^17^. This finding is related to the fact that a longer duration of treatment is required for the management of MDR-TB (9 months to 2 years) than DS-TB (6 months)^6,35^. Moreover, the treatment options for MDR-TB are very limited and expensive, and sometimes recommended medicines are not easily available, particularly in low- and middle-income countries^36,37^. MDR-TB patients also experience many adverse effects from the second-line medications required for the treatment of MDR-TB, and these adverse effects sometimes prolong hospital stays and incur additional medical expenses^38^. The stigma associated with MDR-TB is also higher than the stigma associated with DS-TB^39^, which sometimes leads patients to job loss^38^. Despite basic MDR-TB services being provided free of charge, this has not been sufficient in mitigating the economic consequences of MDR-TB in Brazil, which showed that 18% of MDR-TB patients are exposed to financial hardship^40^. Despite MDR-TB medications being available in public sectors free of charge, a study done in ten high-burden MDR-TB countries showed that 4.8 out of 17 second-line anti-TB recommended drugs are not available through the public health care system^41^. Therefore, patients are obliged to get treatment from the private sector, where TB medications are not available free of charge. A study conducted in India also showed that 70% of MDR-TB patients preferred treatment in private facilities, in which TB medicines are not available free of charge, and the cost of the drugs is the major treatment expense (37%) among MDR-TB patients^42^. However, detailed information about which drugs and investigations are freely provided in the public sector and whether the study was conducted in public or private health facilities were not contained among the included studies.

Our systematic review and meta-analysis showed that the direct costs incurred for treating MDR-TB patients were higher than the indirect costs. This finding is inconsistent with a systematic review conducted among DS-TB patients, in which only 15% of the total cost was incurred from direct costs^17^. Another systematic review and meta-analysis among DS-TB patients conducted in LICs and LMICs countries revealed that direct cost covers only 40% of the total costs^43^. This could be because the component of the direct costs among MDR-TB patients is significantly affected by the longer duration of the treatment^44^. The second possible reason could be due to longer hospitalization, multiple visits to physicians for diagnosis and investigation, purchasing medications, and looking for a special diet in MDR-TB patients^45^. To cover these expenses, MDR-TB patients used different coping mechanisms, including selling assets, borrowing money, and taking children out of school.

By quantifying the burden of catastrophic costs, the current systematic review and meta-analysis provided important information crucial for patients, policymakers, and researchers for designing appropriate strategies to achieve the WHO End-TB Strategy targets of no TB patients or their households facing catastrophic costs. Several interventions can be applied to eliminate catastrophic costs associated with MDR-TB, including early diagnosis and treatment initiation, creating awareness of the availability of free diagnosis and treatment for MDR-TB at public health facilities, and providing social protection and financial support such as nutritional supplementation, comprehensive health cover, and reimbursement for diagnosis, medication, hospitalization, and transportation costs^43,46–48^. Ensuring the implementation of universal health coverage at all levels is also critical to minimize direct and indirect medical costs and to avoid economic consequences on MDR-TB patients and their families^6,49,50^.

This study has several limitations. First, there needed to be more evidence available for some high MDR-TB burden countries such as Bangladesh, India, Myanmar, Nigeria, Pakistan, Russian Federation, and South Africa, which, together, account for more than two-thirds of the global MDR-TB burden. Second, high heterogeneity among the included studies might affect the interpretation of overall estimates. To overcome this limitation and investigate the source of heterogeneity, we conducted a sub-group analysis. Still, we could not run meta-regression due to the few studies included. Third, factors contributing to catastrophic costs were not assessed in this systematic review as different studies reported different risk factors. Fourth, although most of the included studies used a standard WHO tool, some studies used their tools to measure catastrophic costs, which might over or underestimate the proportion of catastrophic costs. Fifth, the burden of catastrophic cost after MDR-TB treatment completion and patients with XDR-TB were not included in the analysis due to a lack of data, which requires further investigation. As most of the studies didn’t report the MDR-TB treatment administered, it could not limit studies that include patients treated with short-term regimens, which are more relevant in recent times. Lastly, the presence of publication bias may have impacted the representativeness of the findings.

## Conclusion

The burden of catastrophic costs was significantly higher among MDR-TB patients than previously reported for DS-TB patients. MDR-TB patients incurred more expenditure for direct costs than indirect costs. Social protection and financial support for patients and affected families are needed to mitigate the catastrophic consequences of MDR-TB. Finally, we recommend that researchers conduct further studies on the cost-effectiveness of home-based care management of MDR-TB patients and its effect on treatment adherence.

### Supplementary Information

Supplementary Information.

## Data Availability

Data will be available upon request from the corresponding author. All the data supporting the findings of this study are available in the table and figures.

## Abbreviations

CI: Confidence Interval
DR-TB: Drug-resistant Tuberculosis
DS-TB: Drug Susceptible Tuberculosis
HICs: High-Income Countries
HIV: Human Immune Deficiency Virus
LICs: Lower Income Countries
LMICs: Lower Middle Income
MDR-TB: Multi-Drug Resistant Tuberculosis
NOS: Newcastle Ottawa Scale
PRISMA: Preferred Reporting Items for Systematic Reviews and Meta-analysis
RR: Rifampicin Resistant
TB: Tuberculosis
UMICs: Upper Middle-Income Countries
WHO: World Health Organization
XDR: Extensively Drug-Resistant Tuberculosis
