# Supplementary material for: Economic burden of multidrug-resistant tuberculosis on patients and households: a global systematic review and meta-analysis
Source: Sci Rep. 2023 Dec 15;13:22361. doi: 10.1038/s41598-023-47094-9 (PMC10724290; doi:10.1038/s41598-023-47094-9)
Supplement: Supplementary file 1 — Supplementary Information. [file 41598_2023_47094_MOESM1_ESM.docx]

Table of contents

Contents

[**Supplementary file: Table 1: PRISMA 20** 2](#_Toc129785000)

[**Supplementary file**: Table 2: Search strategy 4](#_Toc129785001)

[**Supplementary table 3**: Quality of included studies 7](#_Toc129785002)

# **Supplementary file: Table 1: PRISMA 20**

| **Section and Topic** | **Item #** | **Checklist item** | **Location where item is reported** |
| --- | --- | --- | --- |
| **TITLE** | | |  |
| Title | 1 | Identify the report as a systematic review. | Page 1, line 2 |
| **ABSTRACT** | | |  |
| Abstract | 2 | See the PRISMA 2020 for Abstracts checklist. | Page 2, lines 40-42 |
| **INTRODUCTION** | | |  |
| Rationale | 3 | Describe the rationale for the review in the context of existing knowledge. | Page 4, lines 83-86 |
| Objectives | 4 | Provide an explicit statement of the objective(s) or question(s) the review addresses. | Page 4 lines 106-108 |
| **METHODS** | | |  |
| Eligibility criteria | 5 | Specify the inclusion and exclusion criteria for the review and how studies were grouped for the syntheses. | Pages 6, lines 124-134 |
| Information sources | 6 | Specify all databases, registers, websites, organisations, reference lists and other sources searched or consulted to identify studies. Specify the date when each source was last searched or consulted. | Pages 5, lines 114-117 |
| Search strategy | 7 | Present the full search strategies for all databases, registers and websites, including any filters and limits used. | Page 5, lines 117-122 & Supplementary file: table 2 |
| Selection process | 8 | Specify the methods used to decide whether a study met the inclusion criteria of the review, including how many reviewers screened each record and each report retrieved, whether they worked independently, and if applicable, details of automation tools used in the process. | Pages 6, lines 145-147 |
| Data collection process | 9 | Specify the methods used to collect data from reports, including how many reviewers collected data from each report, whether they worked independently, any processes for obtaining or confirming data from study investigators, and if applicable, details of automation tools used in the process. | Page 6, lines 145-147 |
| Data items | 10a | List and define all outcomes for which data were sought. Specify whether all results that were compatible with each outcome domain in each study were sought (e.g. for all measures, time points, analyses), and if not, the methods used to decide which results to collect. | Page 6, lines 136-140 |
|  | 10b | List and define all other variables for which data were sought (e.g. participant and intervention characteristics, funding sources). Describe any assumptions made about any missing or unclear information. | Page 7, 150-155 |
| Study risk of bias assessment | 11 | Specify the methods used to assess risk of bias in the included studies, including details of the tool(s) used, how many reviewers assessed each study and whether they worked independently, and if applicable, details of automation tools used in the process. | Pages 7, lines 176-177 |
| Effect measures | 12 | Specify for each outcome the effect measure(s) (e.g. risk ratio, mean difference) used in the synthesis or presentation of results. | Page 7, lines 168-169 |
| Synthesis methods | 13a | Describe the processes used to decide which studies were eligible for each synthesis (e.g. tabulating the study intervention characteristics and comparing against the planned groups for each synthesis (item #5)). | Page 7, 162-167 |
|  | 13b | Describe any methods required to prepare the data for presentation or synthesis, such as handling of missing summary statistics, or data conversions. | Page 7, line 178 |
|  | 13c | Describe any methods used to tabulate or visually display results of individual studies and syntheses. | Page 7, lines 168-169 |
|  | 13d | Describe any methods used to synthesize results and provide a rationale for the choice(s). If meta-analysis was performed, describe the model(s), method(s) to identify the presence and extent of statistical heterogeneity, and software package(s) used. | Page 7, lines 162-167 |
|  | 13e | Describe any methods used to explore possible causes of heterogeneity among study results (e.g. subgroup analysis, meta-regression). | Page 7&8, lines 169-176 |
|  | 13f | Describe any sensitivity analyses conducted to assess robustness of the synthesized results. | Page 7, 178-179 |
| Reporting bias assessment | 14 | Describe any methods used to assess risk of bias due to missing results in a synthesis (arising from reporting biases). | Page 7, lines 176-177 |
| Certainty assessment | 15 | Describe any methods used to assess certainty (or confidence) in the body of evidence for an outcome. | Page 7, lines 168-169 |
| **RESULTS** | | |  |
| Study selection | 16a | Describe the results of the search and selection process, from the number of records identified in the search to the number of studies included in the review, ideally using a flow diagram. | Page 8, lines 188-192 and Fig 1 |
|  | 16b | Cite studies that might appear to meet the inclusion criteria, but which were excluded, and explain why they were excluded. | NA |
| Study characteristics | 17 | Cite each included study and present its characteristics. | Page 7 line 191 |
| Risk of bias in studies | 18 | Present assessments of risk of bias for each included study. | Page 10, lines 232-233 and **S**: Fig 4 |
| Results of individual studies | 19 | For all outcomes, present, for each study: (a) summary statistics for each group (where appropriate) and (b) an effect estimate and its precision (e.g. confidence/credible interval), ideally using structured tables or plots. | Page 9, Lines 209-213, and Figure 3 |
| Results of syntheses | 20a | For each synthesis, briefly summarise the characteristics and risk of bias among contributing studies. | Page 10, lines 232-233 and **S**: Fig 4 |
|  | 20b | Present results of all statistical syntheses conducted. If meta-analysis was done, present for each the summary estimate and its precision (e.g. confidence/credible interval) and measures of statistical heterogeneity. If comparing groups, describe the direction of the effect. | Page 9, Lines 209-213, and Figure 3 |
|  | 20c | Present results of all investigations of possible causes of heterogeneity among study results. | Page 9, Lines 215-221  And Table 3 |
|  | 20d | Present results of all sensitivity analyses conducted to assess the robustness of the synthesized results. | Page 10, 235-237, and **S:** Fig 5 |
| Reporting biases | 21 | Present assessments of risk of bias due to missing results (arising from reporting biases) for each synthesis assessed. | Page 10, lines 232-233 and **S**: Fig 4 |
| Certainty of evidence | 22 | Present assessments of certainty (or confidence) in the body of evidence for each outcome assessed. | Page 9, Lines 209-213, Fig 3, **Supplementary file** |
| **DISCUSSION** | | |  |
| Discussion | 23a | Provide a general interpretation of the results in the context of other evidence. | Page 10&12, lines 239-279 |
|  | 23b | Discuss any limitations of the evidence included in the review. | Page 12, lines 291-294 |
|  | 23c | Discuss any limitations of the review processes used. | Page 12 & 13, lines 294-306 |
|  | 23d | Discuss the implications of the results for practice, policy, and future research. | Pages 12, lines 280-290 |
| **OTHER INFORMATION** | | |  |
| Registration and protocol | 24a | Provide registration information for the review, including the register name and registration number, or state that the review was not registered. | Page 2, lines 41-42 |
|  | 24b | Indicate where the review protocol can be accessed, or state that a protocol was not prepared. | Page 2, lines 41-42 |
|  | 24c | Describe and explain any amendments to information provided at registration or in the protocol. | NA |
| Support | 25 | Describe sources of financial or non-financial support for the review, and the role of the funders or sponsors in the review. | Page 14, lines 324-328 |
| Competing interests | 26 | Declare any competing interests of review authors. | Page 14, line 330 |
| Availability of data, code and other materials | 27 | Report which of the following are publicly available and where they can be found: template data collection forms; data extracted from included studies; data used for all analyses; analytic code; any other materials used in the review. | Page 14. Line 336-337 |

#

# **Supplementary file**: Table 2: Search strategy

| Database | Search terms | Output |
| --- | --- | --- |
| **Embase** | | |
| 1 | ("multidrug-resistant* tuberculosis" or "multidrug-resistant* TB" or extensively drug-resistant* or "drug-resistant* tuberculosis" or "drug-resistant* TB" or "MDR-TB" or "XDR-TB" or "DR-TB").mp. [mp=title, abstract, heading word, drug trade name, original title, device manufacturer, drug manufacturer, device trade name, keyword heading word, floating subheading word, candidate term word] | 19,584 |
| 2 | (Drug-resistant tuberculosis or multidrug-resistant tuberculosis or extensively drug-resistant tuberculosis).mp. [mp=title, abstract, heading word, drug trade name, original title, device manufacturer, drug manufacturer, device trade name, keyword heading word, floating subheading word, candidate term word] | 15,305 |
| 3 | ("costs" or "loss" or "sales" or "loan" or "economics" or "finance*" or "cost*" or "expense" or "expenditure*" or "payment" or "impoverishment").mp. [mp=title, abstract, heading word, drug trade name, original title, device manufacturer, drug manufacturer, device trade name, keyword heading word, floating subheading word, candidate term word] | 3,151,980 |
| 4 | 1 OR 2 | 19,584 |
| 5 | 3 AND 4 | 2,666 |
| **Medline** | | |
| #1 | ("multidrug-resistant* tuberculosis" or "multidrug-resistant* TB" or extensively drug resistant* or "drug resistant* tuberculosis" or "drug resistant* TB" or "MDR-TB" or "XDR-TB" or "DR-TB").mp. [mp=title, book title, abstract, original title, name of substance word, subject heading word, floating sub-heading word, keyword heading word, organism supplementary concept word, protocol supplementary concept word, rare disease supplementary concept word, unique identifier, synonyms] | 12,632 |
| #2 | (Drug-resistant tuberculosis or multidrug-resistant tuberculosis or extensively drug-resistant tuberculosis).mp. [mp=title, book title, abstract, original title, name of substance word, subject heading word, floating sub-heading word, keyword heading word, organism supplementary concept word, protocol supplementary concept word, rare disease supplementary concept word, unique identifier, synonyms] | 7,847 |
| #3 | ("costs" or "loss" or "sales" or "loan" or "economics" or "finance*" or "cost*" or "expense" or "expenditure*" or "payment" or "impoverishment").mp. [mp=title, book title, abstract, original title, name of substance word, subject heading word, floating sub-heading word, keyword heading word, organism supplementary concept word, protocol supplementary concept word, rare disease supplementary concept word, unique identifier, synonyms] | 2,337,017 |
| #4 | 1 OR 2 | 12632 |
| #5 | 3 AND 4 | 1,457 |
| **CINAHL Plus with Full Text** | | |
| #1 | TI "multidrug-resistant* tuberculosis" OR "multidrug-resistan* TB" OR extensively drug resistan* OR "drug resistan* tuberculosis" OR "drug resistan* TB" OR "MDR-TB" OR "XDR-TB" OR "DR-TB" | 1,453 |
| #2 | TI "costs" or "loss" or "sales" or "loan" or "economics" or "financ*" or "cost*" or "expense" or "expenditure*" or "payment" or "impoverishment" | 129,126 |
| #3 | 1 AND 2 | 47 |
| **Web of Science** | | |
| #1 | TI =("multidrug-resistant* tuberculosis" OR "multidrug-resistan* TB" OR extensively drug resistan* OR "drug resistan* tuberculosis" OR "drug resistan* TB" OR "MDR-TB" OR "XDR-TB" OR "DR-TB") | 6,202 |
| #2 | TI=("costs" or "loss" or "sales" or "loan" or "economics" or "financ*" or "cost*" or "expense" or "expenditure*" or "payment" or "impoverishment") | 849,256 |
| #3 | 1 AND 2 | 155 |
|  | **Scopus** |  |
| #1 | TITLE-ABS-KEY ( "multidrug-resistan* tuberculosis" OR "multidrug-resistan* tb" OR extensively AND drug AND resistan* OR "drug resistan* tuberculosis" OR "drug resistan* tb" OR "MDR-tb" OR "XDR-tb" OR "dr-tb" ) | 17,972 |
| #2 | ( TITLE-ABS-KEY ( "multidrug-resistan*tuberculosis" OR "multidrug-resistan* tb" OR extensively AND drug AND resistan* OR "drug resistan* tuberculosis" OR "drug resistan* tb" OR "MDR-tb" OR "XDR-tb" OR "dr-tb" ) AND TITLE-ABS-KEY ( "costs" OR "loss" OR "sales" OR "loan" OR "economics" OR "financ*" OR "cost*" OR "expense" OR "expenditure*" OR "payment" OR "impoverishment" ) ) | 7,465,718 |
| #3 | 1 AND 2 | 2,241 |

# **Supplementary Table 3**: Quality of included studies

|  | | |  | |  |  |  |  |  |  |  |  |
| --- | --- | --- | --- | --- | --- | --- | --- | --- | --- | --- | --- | --- |
| **S.No** | **Study name** | **Selection** | | | | | | **Comparators** | **Outcome** | | **Total (10%)** | **Decision** |
|  |  | **Representativeness (*)** | | **Sample size (*)** | | **Non-respondents (*)** | **Ascertainment of exposure (*)** | **Comparability (**)** | **Assessment of outcome (**)** | **Statistical tests (*)** |  |  |
| 1 | Nhung (2018) | 1 | | 0 | | 1 | 1 | 0 | 1 | 0 | 4 | Poor-quality |
| 2 | Collins (2021) | 1 | | 1 | | 1 | 1 | 0 | 1 | 0 | 5 | Moderate quality |
| 3 | Phonenaly (2020) | 1 | | 1 | | 0 | 1 | 0 | 1 | 0 | 4 | Poor-quality |
| 4 | Yun (2020) | 1 | | 1 | | 1 | 0 | 0 | 0 | 0 | 3 | Poor-quality |
| 5 | Ahmad (2018) | 0 | | 1 | | 1 | 1 | 2 | 1 | 1 | 7 | Moderate quality |
| 6 | Winters (2020) | 0 | | 0 | | 0 | 1 | 0 | 1 | 0 | 2 | Poor-quality |
| 7 | Kaswa (2021) | 1 | | 1 | | 1 | 1 | 0 | 1 | 0 | 5 | Moderate quality |
| 8 | Tomeny (2020) | 0 | | 1 | | 1 | 1 | 0 | 1 | 0 | 4 | Poor-quality |
| 9 | Andrew (2022) | 1 | | 1 | | 1 | 1 | 0 | 1 | 0 | 5 | Moderate quality |
| 10 | Debora (2018) | 1 | | 0 | | 1 | 1 | 0 | 1 | 0 | 4 | Poor-quality |

|  | | **Quality assessment for a cohort study** | | | | | | | | | | |
| --- | --- | --- | --- | --- | --- | --- | --- | --- | --- | --- | --- | --- |
| **S.N** | **Study name** | | **Selection** | | | | **Comparators** | **Outcomes** | | | **Total (9%)** | **Decision** |
|  |  |  | **Representativeness (*)** | **Selection of non-exposed cohort (*)** | **Ascertainment of exposure (*)** | **Demonstration of outcome is not present at the start (*)** | **Comparability (**) of cohorts on the design or analysis** | **Assessment of outcome (*)** | **Follow-up long enough (*)** | **Adequacy of follow-up (*)** |  |  |
| 11 | TOM (2014) | | 1 | 1 | 0 | 1 | 0 | 1 | 1 | 1 | 6 | High |

Poor quality Studies: 0 to 4 points, Moderate quality Studies: 5-7 points, 8-9 points: high quality studies
